# A Phase 1/2, Dose-Escalation Trial of Deferasirox for the Treatment of Iron Overload in *HFE*-Related Hereditary Hemochromatosis

**DOI:** 10.1002/hep.23879

**Published:** 2010-11

**Authors:** Pradyumna Phatak, Pierre Brissot, Mark Wurster, Paul C Adams, Herbert L Bonkovsky, John Gross, Peter Malfertheiner, Gordon D McLaren, Claus Niederau, Alberto Piperno, Lawrie W Powell, Mark W Russo, Ulrich Stoelzel, Wolfgang Stremmel, Louis Griffel, Nicola Lynch, Yiyun Zhang, Antonello Pietrangelo

**Affiliations:** Rochester General HospitalRochester, NY; Pontchaillou University Hospital, Institut National de la Santé et de la Recherche Médicale U-991 and CIC 0203Rennes, France; Ohio State University Medical CenterColumbus, OH; University HospitalLondon, Ontario, Canada; Cannon Research Center at Carolinas Health Care SystemCharlotte, NC; Mayo ClinicRochester, MN; Medizinische Fakultat der Otto-von-Guericke-Universität MagdeburgMagdeburg, Germany; Chao Family Comprehensive Cancer Center, University of CaliforniaIrvine, CA, and VA Medical Center, Long Beach, CA; St Josef Hospital, University of EssenOberhausen, Germany; Department of Clinical Medicine and Prevention, University of Milano-BicoccaMonza, Italy; Royal Brisbane and Women's Hospital and the University of QueenslandBrisbane, Australia; Carolinas Medical CenterCharlotte, NC; Klinikum ChemnitzChemnitz, Germany; University HospitalHeidelberg, Germany; Novartis Pharmaceuticals Corp.East Hanover, NJ; University of Modena and Reggio EmiliaModena, Italy

## Abstract

Hereditary hemochromatosis (HH) is characterized by increased intestinal iron absorption that may result in iron overload. Although phlebotomy is widely practiced, it is poorly tolerated or contraindicated in patients with anemias, severe heart disease, or poor venous access, and compliance can vary. The once-daily, oral iron chelator, deferasirox (Exjade) may provide an alternative treatment option. Patients with HH carrying the *HFE* gene who were homozygous for the Cys282Tyr mutation, serum ferritin levels of 300-2000 ng/mL, transferrin saturation ≥45%, and no known history of cirrhosis were enrolled in this dose-escalation study to characterize the safety and efficacy of deferasirox, comprising a core and an extension phase (each 24 weeks). Forty-nine patients were enrolled and received starting deferasirox doses of 5 (n = 11), 10 (n = 15), or 15 (n = 23) mg/kg/day. Adverse events were generally dose-dependent, the most common being diarrhea, headache, and nausea (n = 18, n = 10, and n = 8 in the core and n = 1, n = 1, and n = 0 in the extension, respectively). More patients in the 15 mg/kg/day than in the 5 or 10 mg/kg/day cohorts experienced increases in alanine aminotransferase and serum creatinine levels during the 48-week treatment period; six patients had alanine aminotransferase >3× baseline and greater than the upper limit of normal range, and eight patients had serum creatinine >33% above baseline and greater than upper limit of normal on two consecutive occasions. After receiving deferasirox for 48 weeks, median serum ferritin levels decreased by 63.5%, 74.8%, and 74.1% in the 5, 10, and 15 mg/kg/day cohorts, respectively. In all cohorts, median serum ferritin decreased to <250 ng/mL. *Conclusion:* Deferasirox doses of 5, 10, and 15 mg/kg/day can reduce iron burden in patients with HH. Based on the safety and efficacy results, starting deferasirox at 10 mg/kg/day appears to be most appropriate for further study in this patient population. (Hepatology 2010)

Hereditary hemochromatosis (HH) is one of the most common genetic diseases, and most patients are homozygous for the HH gene *HFE* Cys282Tyr (C282Y) mutation. Homozygosity for C282Y may be associated with excessive absorption of dietary iron in some cases. If untreated, progressive iron accumulation in key organs leads to toxicity, resulting in tissue damage and organ dysfunction.^1^–^3^ Patients with HH and cirrhosis are also at significant risk of developing hepatocellular carcinoma.^4^ Iron overload can be readily managed in most patients by therapeutic phlebotomy. However, patients with underlying anemias, severe heart disease, or reduced venous access may not tolerate this form of treatment.^5^ In addition, compliance may also be variable over time^6^ as a result of the inconvenience of frequent clinic visits and the discomfort associated with the procedure.

The once-daily oral iron chelator deferasirox (Exjade; Novartis Pharma AG, Basel, Switzerland) is indicated for the treatment of chronic iron overload due to frequent blood transfusions at approved doses of 10-40 mg/kg body weight/day. Clinical studies have demonstrated the efficacy, safety, and tolerability of deferasirox in patients with a variety of conditions associated with transfusional iron overload.^7^–^11^ Studies reporting iron chelation therapy in patients with primary (nontransfusional) iron overload are limited, but data are encouraging.^12^–^16^ As patients with HH generally have a lower relative iron burden than those with transfusional iron overload and do not accumulate iron at the same rate, the safety and efficacy of iron chelation therapy in this setting requires further evaluation. The current study is the first clinical trial to assess the safety and efficacy of deferasirox therapy (at the lower end of the approved dose range for patients with transfusional iron overload) in patients with HH who required de-ironing based on elevated serum ferritin levels.

## Patients and Methods

### Patient Recruitment

The study was conducted across 18 sites in six countries (United States, France, Germany, Italy, Australia, and Canada). This was an open-label, multicenter, dose-escalation study designed to characterize the safety and efficacy of deferasirox using four dose levels (5, 10, 15, and 20 mg/kg/day). Male and female patients (aged ≥18 years) with homozygosity for the C282Y HFE mutation (as documented by molecular diagnostic testing) and iron overload (as shown by serum ferritin levels of 300-2000 ng/mL and serum transferrin saturation ≥45%) were included in the study. Key exclusion criteria were: males with hemoglobin concentrations <13 g/dL or females with hemoglobin concentrations <12 g/dL; treatment with phlebotomy within 2 weeks of screening; history of blood transfusion during the 6 months prior to study entry; current or previous treatment with deferiprone (Ferriprox; Apotex, Inc., Toronto, ON, Canada) or deferasirox; serum creatinine levels above the upper limit of the normal range (ULN); alanine aminotransferase (ALT) levels of ≥2× ULN; or known diagnosis of liver cirrhosis. All patients provided written, informed consent at the start of the core and extension periods of the study. The study was conducted in accordance with Good Clinical Practice guidelines and the Declaration of Helsinki.

### Study Design

After all eligibility criteria had been confirmed, at least eight patients were planned to be enrolled in each of the four dose levels in a sequential manner. Opening of the next dose level (dose escalation) followed a safety review by an independent safety monitoring committee; this was conducted once the sixth patient enrolled in that stratum had been treated for 4 weeks. Dose escalation was guided by a Bayesian logistic regression model^17^,^18^ for the most common severe adverse events (AEs; abdominal pain, nausea, vomiting, diarrhea, rash, increased serum creatinine, and increased aminotransferases) and clinical review. The recommended dose level at completion of the core study was based on an overall assessment of safety and efficacy, taking into account all tolerability and serum ferritin data collected at each dose level tested. At the end of the core period of 24 weeks, patients were invited to enter an extension study for a further 24 weeks of therapy with deferasirox. Patients who chose not to continue the study did not have to provide a reason.

### Assessments

The primary objective of the core study was to assess the safety of deferasirox therapy, with continued assessment in the 24-week extension phase. Safety assessments consisted of monitoring and recording all AEs (including serious AEs), the regular monitoring of biochemistry, hematology, urinalysis, electrocardiogram, audiometry, and ocular examination data. The effect of deferasirox therapy on serum ferritin levels was assessed as a secondary objective. If serum ferritin levels fell to <100 ng/mL at any visit, deferasirox was interrupted until levels rose to >300 ng/mL. Serum ferritin, serum iron, serum transferrin, calculated total iron-binding capacity, and transferrin saturation parameters were measured at screening and at each study visit.

### Statistical Methods

All patients who received at least one dose of study drug, and had at least one post-baseline safety assessment, were included in the safety population. The per-protocol population comprised patients from the safety population who had no major protocol violations and was used for selected efficacy analyses. Median decrease in serum ferritin levels in the core and extension studies were analyzed based on the signed-rank test for the three respective dose cohorts. Changes in serum ferritin levels among the three dose cohorts from baseline to end of study were evaluated by performing an analysis of covariance, where the baseline serum ferritin measure was fitted as a continuous covariate. A longitudinal analysis was also performed by a linear mixed-effects model for serum ferritin. The statistical model for the dose escalation of deferasirox was based on a two-parameter logistic model.

## Results

### Patient Characteristics

A total of 49 Caucasian patients with HH were enrolled in the core study (Table 1) and received starting doses of deferasirox of either 5 (n = 11), 10 (n = 15), or 15 (n = 23) mg/kg/day for at least 24 weeks. The study was initiated on August 23, 2006, and completed on October 2, 2008, and March 19, 2009, for the core and extension study, respectively.

**Table 1:** Patient Demographics and Characteristics at Baseline

Characteristic	5 mg/kg/day (n = 11)	10 mg/kg/day (n = 15)	15 mg/kg/day (n = 23)	All Patients (n = 49)
Mean age ± SD, years	55.8 ± 12.8	47.8 ± 10.3	49.8 ± 16.4	50.6 ± 14.0
Male:female	9:2	11:4	13:10	33:16
Mean time since diagnosis ± SD, years	6.6 ± 7.0	1.3 ± 2.0	2.6 ± 3.8	3.1 ± 4.7
Median serum ferritin (range), ng/mL*	512 (376–1729)	859 (447–1792)	634 (357–1600)	645 (357–1792)
Mean transferrin saturation ± SD, %*	78.1 ± 17.4	81.0 ± 12.7	74.1 ± 15.4	77.2 ± 15.2

* Based on the per-protocol population.

After 49 patients had been enrolled in the study and 23 patients had been treated at the 15 mg/kg/day dose level, further dose escalation was stopped. The estimated mean rate of the most common severe AEs at 20 mg/kg/day was determined to be 34.9% compared with 17.4% at the 15 mg/kg/day dose level, suggesting that dose escalation should not continue beyond 15 mg/kg/day. All 49 patients were included in the safety analysis and 48 patients were included in the efficacy analysis (per-protocol; one patient was excluded due to clinical evidence of active hepatitis B/C).

Thirty-seven (75.5%) patients completed the core study (10 [90.9%], 11 [73.3%], and 16 [69.6%] patients in the 5, 10, and 15 mg/kg/day cohorts, respectively). Twenty-six patients decided to continue into the extension study; 23 (88.5%) patients (nine [100%], six [100%], eight [72.7%] patients in the 5, 10, and 15 mg/kg/day cohorts, respectively) completed the 24-week extension.

### Patient Discontinuations

**Figure 1:**
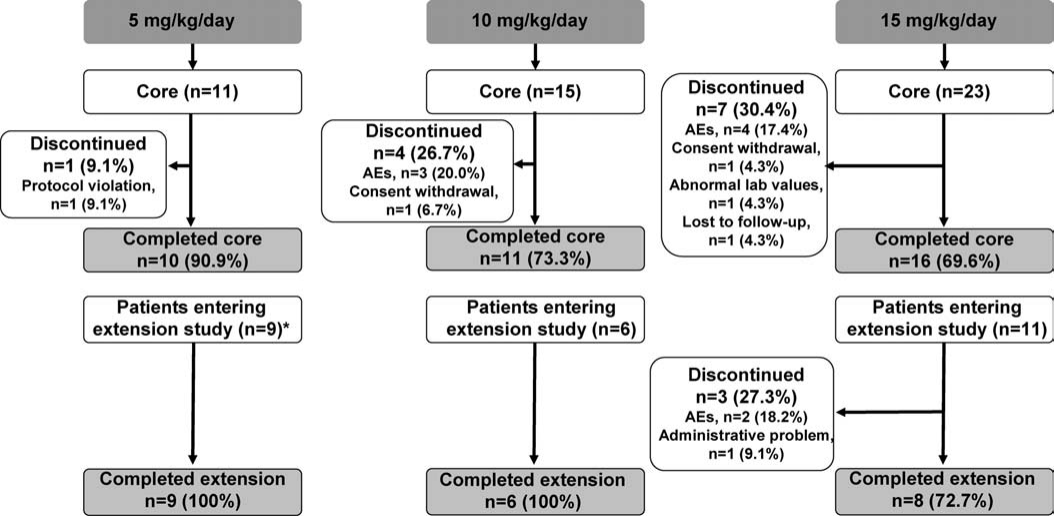
Patient disposition in the core and extension study. *The 5 mg/kg/day dose reduced serum ferritin in only three patients; the other six patients in this dose cohort received 10 mg/kg/day at the start of the extension (all but one patient experienced serum ferritin levels <100 ng/mL when receiving 10 mg/kg/day).

During the core and extension study, 15 patients discontinued treatment. Reasons for discontinuation in the core study were: protocol deviation (n = 1) in the 5 mg/kg/day cohort; AEs (n = 3) and consent withdrawal (n = 1) in the 10 mg/kg/day cohort; and AEs (n = 4), consent withdrawal, abnormal laboratory value and loss to follow-up (n = 1 for each) in the 15 mg/kg/day cohort (Fig. 1). In the extension study, three patients discontinued in the 15 mg/kg/day cohort primarily due to AEs (n = 2; Fig. 1). AEs leading to discontinuation were ALT elevation, increased bilirubin levels (attributed to preexisting Gilbert's syndrome; peak total bilirubin of 104.3 μmol/L) and diarrhea/nausea/backache/fatigue in the core 10 mg/kg/day cohort (n = 1 for each); severe rash, serum creatinine elevation, nausea, and ALT elevation in the core 15 mg/kg/day cohort (n = 1 for each); and diarrhea and increased levels of serum aminotransferases in the extension 15 mg/kg/day cohort (n = 1 for both).

### Safety Assessment

The most common investigator-reported AEs after the start of deferasirox treatment (≥10% in all patients) during the core study were diarrhea, headache, nausea, and abdominal pain (Table 2). Common AEs suspected to be drug-related (≥10% in all patients) were diarrhea, nausea, and abdominal pain (Table 3). There were no serious AEs or deaths reported by the investigators. In the extension study, the incidence of newly reported AEs and drug-related AEs was reduced compared with the core study (Tables 2 and 3).

**Table 2:** Most Common AEs (≥10% of All Patients in Core or Extension)

	n (%)							
	
	5 mg/kg/day		10 mg/kg/day		15 mg/kg/day		All Patients	
				
Adverse Event	Core (n = 11)	Ext (n = 9)	Core (n = 15)	Ext (n = 6)	Core (n = 23)	Ext (n = 11)	Core (n = 49)	Ext (n = 26)
Diarrhea	2 (18.2)	0	6 (40.0)	0	10 (43.5)	1 (9.1)	18 (36.7)	1 (3.8)
Headache	1 (9.1)	0	2 (13.3)	0	7 (30.4)	1 (9.1)	10 (20.4)	1 (3.8)
Nausea	0	0	2 (13.3)	0	6 (26.1)	0	8 (16.3)	0
Abdominal pain	0	0	3 (20.0)	0	4 (17.4)	0	7 (14.3)	0
Increased serum creatinine*	0	1 (11.1)	3 (20.0)	1 (16.7)	4 (17.4)	1 (9.1)	7 (14.3)	3 (11.5)
Back pain	1 (9.1)	1 (11.1)	4 (26.7)	0	2 (8.7)	0	7 (14.3)	1 (3.8)
Increased ALT*	0	1 (11.1)	3 (20.0)	0	3 (13.0)	0	6 (12.2)	1 (3.8)
Rash	1 (9.1)	0	1 (6.7)	1 (16.7)	4 (17.4)	0	6 (12.2)	1 (3.8)
Flatulence	2 (18.2)	0	1 (6.7)	0	2 (8.7)	0	5 (10.2)	0
Nasopharyngitis	1 (9.1)	0	1 (6.7)	0	3 (13.0)	0	5 (10.2)	0
Fatigue	1 (9.1)	1 (11.1)	1 (6.7)	1 (16.7)	2 (8.7)	1 (9.1)	4 (8.2)	3 (11.5)
Arthralgia	1 (9.1)	0	2 (13.3)	2 (33.3)	1 (4.3)	1 (9.1)	4 (8.2)	3 (11.5)

* Reported based on clinical signs or symptoms, considered clinically significant by investigator or requiring therapy as a result.

**Table 3:** Most Common Drug-Related AEs (≥10% of All Patients in Core or Extension)

	n (%)							
	
	5 mg/kg/day		10 mg/kg/day		15 mg/kg/day		All Patients	
				
Adverse Event	Core (n = 11)	Ext (n = 9)	Core (n = 15)	Ext (n = 6)	Core (n = 23)	Ext (n = 11)	Core (n = 49)	Ext (n = 26)
Diarrhea	1 (9.1)	0	4 (26.7)	0	9 (39.1)	1 (9.1)	14 (28.6)	1 (3.8)
Increased serum creatinine*	0	1 (11.1)	3 (20.0)	1 (16.7)	4 (17.4)	1 (9.1)	7 (14.3)	3 (11.5)
Nausea	0	0	2 (13.3)	0	4 (17.4)	0	6 (12.2)	0
Abdominal pain	0	0	2 (13.3)	0	3 (13.0)	0	5 (10.2)	0
Increased ALT*	0	1 (11.1)	3 (20.0)	0	2 (8.7)	0	5 (10.2)	1 (3.8)

* Reported based on clinical signs or symptoms, considered clinically significant by investigator or required therapy as a result.

Over the entire study (48 weeks), more patients in the 15 mg/kg/day cohort experienced increases in ALT or aspartate aminotransferase (AST) levels >3× baseline and greater than ULN than the lower dose cohorts (Table 4). All eight patients who had ALT >3× baseline and greater than ULN had their dose interrupted; four discontinued the study, three did not restart, and one was observed until the end of the core study but did not enter the extension. Of these patients with ALT events, only one patient had an increase in bilirubin greater than ULN.

**Table 4:** Serum Creatinine and Liver Function Tests

Core and Extension*	5 mg/kg/day (n = 11)	10 mg/kg/day (n = 15)	15 mg/kg/day (n = 23)	All Patients (n = 49)
Serum creatinine, increase >33% above baseline and >ULN at 2 consecutive visits, n (%)	0	5 (33.3)	8 (34.8)	13 (26.5)
AST				
Increase >3× baseline and >ULN, n (%)	1 (9.1)	2 (13.3)	5 (21.7)	8 (16.3)
Increase >5× baseline and >ULN, n (%)	0	0	5 (21.7)	5 (2.0)
ALT				
Increase >3× baseline and >ULN, n (%)	1 (9.1)	1 (6.7)	6 (26.1)	8 (16.3)
Increase >5× baseline and >ULN, n (%)	0	0	5 (21.7)	5 (2.0)

* Patients were counted only once in the core or extension.

In the core study, two patients had ALT levels >5× ULN (both in the 15 mg/kg/day cohort; one patient had elevated levels at baseline), and 11 patients had serum creatinine levels >33% above baseline and greater than ULN on two consecutive occasions (five patients in the 10 mg/kg/day cohort and six patients in the 15 mg/kg/day cohort). In the extension study, one patient experienced an increase in ALT levels >5× ULN (15 mg/kg/day cohort; levels were elevated at baseline), and seven patients had serum creatinine levels >33% above baseline and greater than ULN on two consecutive occasions (two patients in the 10 mg/kg/day cohort and five patients in the 15 mg/kg/day cohort). Patients were managed with dose decreases and/or interruptions.

### Serum Ferritin and Transferrin Saturation

**Figure 2:**
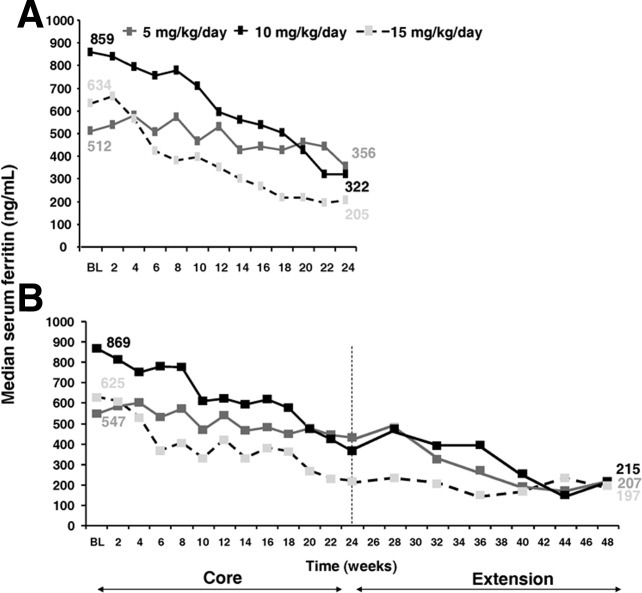
Median serum ferritin in (A) patients enrolled in the core study and (B) patients who completed the core and continued into the extension study. BL, baseline. Note that the 5 mg/kg/day dose reduced serum ferritin in three patients; the other six patients in this dose cohort received 10 mg/kg/day at the start of the extension (all but one patient experienced serum ferritin levels <100 ng/mL when receiving 10 mg/kg/day).

Of the patients completing the core and extension study, 26 of 37 (70.3%) and 21 of 23 (91.3%) achieved serum ferritin levels of <500 ng/mL. Of the 23 patients completing 48 weeks of deferasirox treatment, 15 (65.2%) had serum ferritin levels of <250 ng/mL by the end of the extension study. Four (36.4%), two (13.3%), and eight (36.4%) patients (per-protocol) had serum ferritin levels of <100 ng/mL in the 5, 10, and 15 mg/kg/day cohorts, respectively, at some point during the 48-week treatment period. Reductions in serum ferritin levels were observed in the core study across all three dose cohorts with a median decrease of 31.1% (*P* = 0.08 [nonsignificant]), 52.8% (*P* = 0.0005), and 55.4% (*P* < 0.0001) in the 5, 10, and 15 mg/kg/day cohorts, respectively. The time course of the serum ferritin decrease was dose-dependent (Fig. 2A). In the patients who continued deferasirox therapy in the extension study, serum ferritin levels continued to decrease with a median reduction of 63.5% (*P* = 0.002), 74.8% (*P* < 0.0001), and 74.1% (*P* = 0.0004) from the core baseline in the 5, 10, and 15 mg/kg/day cohorts, respectively (Fig. 2B). However, it should be noted that six of the nine patients in the 5 mg/kg/day cohort received an increased dose of 10 mg/kg/day during the extension. Changes in serum ferritin levels at the end of the core study were not statistically different at a 0.05 level between the three dose cohorts. Longitudinal analysis over the core and extension study showed that the reduction in serum ferritin from baseline levels was significantly greater in the 10 and 15 mg/kg/day cohorts compared with the 5 mg/kg/day cohort (*P* < 0.05 for both). There was no significant difference in serum ferritin level decrease between the 10 and 15 mg/kg/day cohorts. Reductions in transferrin saturation were also observed in the core study across all three dose cohorts, with a mean decrease of 6.4%, 10.7%, and 2.1% in the 5, 10, and 15 mg/kg/day cohorts, respectively. In the patients who continued deferasirox therapy in the extension study, transferrin saturation continued to decrease in the 10 and 15 mg/kg/day cohorts, resulting in a mean decrease from baseline to the end of the extension by 14.4% and 12.1%, respectively. However, the overall mean change from baseline in the 5 mg/kg/day cohort was +0.9%.

### Relationship Between Serum Ferritin and Hepatic/Renal Parameters

**Figure 3:**
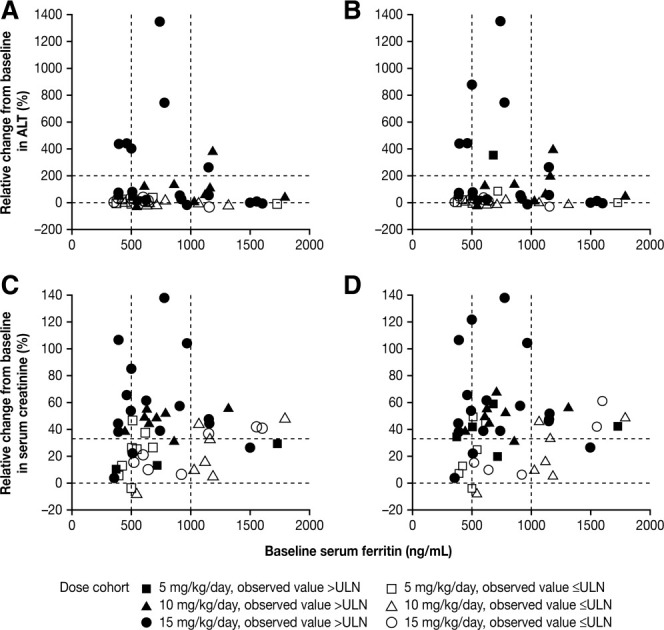
Scatter plots of baseline serum ferritin versus worst ALT relative change (%) during (A) 24 weeks of treatment and (B) 48 weeks of treatment; and worst serum creatinine relative change (%) during (C) 24 weeks of treatment and (D) 48 weeks of treatment, by dose cohort and ULN.

Subsequent analyses were performed to evaluate the relationship between baseline serum ferritin levels and changes in ALT and serum creatinine levels. These analyses suggested that ALT and serum creatinine increases may have been associated with relative deferasirox doses that were too high compared with baseline serum ferritin levels (Fig. 3).

**Figure 4:**
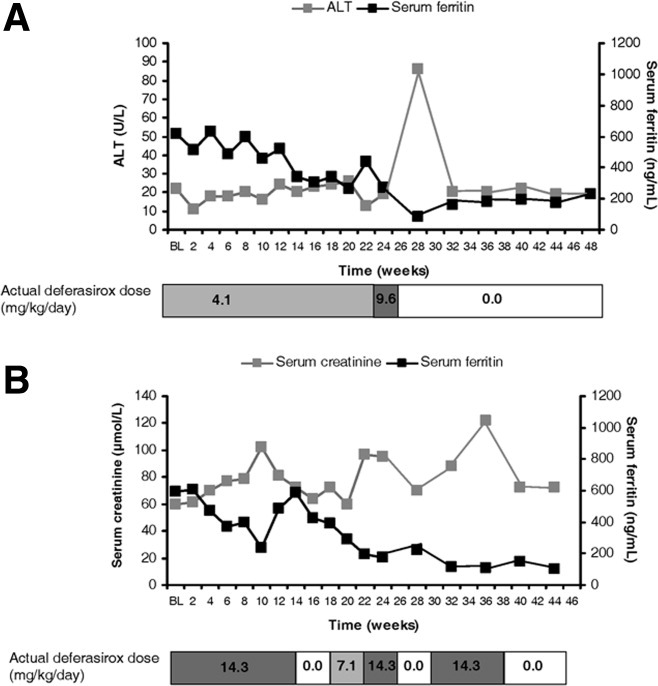
Individual patient examples of the relationship between serum ferritin and (A) ALT and (B) serum creatinine.

Five of the six patients in the 15 mg/kg/day cohort who had ALT levels >3× baseline and greater than ULN had relatively low serum ferritin levels at baseline (range 356-740 ng/mL). Two of these patients had sharp rises in ALT concentration associated with rapid reductions in serum ferritin to 70 and 108 ng/mL at week 6 and 36, respectively. The sixth patient had a baseline serum ferritin value of 1083 ng/mL, but experienced a rapid reduction at week 4 to 758 ng/mL. The patient in the 5 mg/kg/day cohort who had an ALT elevation of >3× baseline and greater than ULN had a baseline serum ferritin value of 740 ng/mL, and at the time of reaching the ALT elevation, had experienced a reduction in serum ferritin levels to 83 ng/mL following an increase in deferasirox dose (Fig. 4A). The patient in the 10 mg/kg/day cohort with increased ALT levels >3× baseline and greater than ULN did not show any distinct relationships between serum ferritin and ALT increases.

Four of the five patients in the 10 mg/kg/day cohort who had serum creatinine levels >33% above baseline and greater than ULN at two consecutive visits had relatively low baseline serum ferritin levels (range 587-743 ng/mL); the remaining patient had a baseline serum ferritin value of 1316 ng/mL, but experienced a rapid reduction to 174 ng/mL by week 14. Baseline serum ferritin levels were similarly low in six of the eight patients in the 15 mg/kg/day cohort (range 490-896 ng/mL); the other two patients had rapid reductions in serum ferritin values from 1018 to 494 ng/mL at week 4 and from 1186 to 523 ng/mL at week 24, respectively. Overall in both dose cohorts, initial notable increases in serum creatinine levels corresponding with reductions in serum ferritin were observed early (within 4-6 weeks) in seven patients. The remaining six patients had serum creatinine increases by the end of the 24-week core study. One patient in the 15 mg/kg/day cohort had notable serum creatinine increases on several occasions in association with reductions in serum ferritin. On each of these occasions, the serum creatinine levels returned to baseline levels after deferasirox was temporarily discontinued (Fig. 4B).

## Discussion

This is the first clinical trial to demonstrate the safety and efficacy of deferasirox in a population of patients with C282Y homozygous HH and primary iron overload. There was higher enrollment of males than females as expected, because female C282Y homozygotes tend to have lower levels of iron overload than male homozygotes.^19^ Although phlebotomy is the first line of treatment for patients with HH, these data indicate that chelation therapy with deferasirox may offer an alternative approach to the management of iron accumulation for patients who are unwilling or unable to comply with de-ironing by phlebotomy. The results from the 24-week core study demonstrated that the AE profile was consistent with the known safety profile of deferasirox; all described gastrointestinal, renal, and hepatic AEs are known, labeled, and are clinically manageable with regular patient monitoring as reported in patients with transfusion-induced iron overload. The frequency of AEs was dose-dependent, being highest in the 15 mg/kg/day patient cohort; the estimated incidence of AEs at 20 mg/kg/day precluded dose escalation beyond 15 mg/kg/day. AEs generally occurred early during treatment as shown by the higher frequency reported during the core compared with the extension study. However, these data could be limited by the potential for self-selection of patients; for example, it is possible that patients experiencing AEs in the core phase of the study decided not to continue in the extension, reflecting the reduced frequency of AEs reported.

Deferasirox doses of 5, 10, and 15 mg/kg/day reduced serum ferritin levels in this population of patients with HH, although it should be noted that six of the nine patients in the 5 mg/kg/day cohort who entered the extension study were dose escalated to 10 mg/kg/day. The percentage change in serum ferritin level was greater in the 10 and 15 mg/kg/day cohorts compared with the 5 mg/kg/day cohort and similar in the 10 and 15 mg/kg/day cohorts. When considered together with the safety results, where a higher frequency of AEs were reported in the 15 mg/kg/day cohort, a starting dose of 10 mg/kg/day appears to be the most appropriate in this population of patients. This is a starting dose lower than that generally recommended for patients with transfusional iron overload.^7^,^10^,^20^

Subsequent analyses of these data suggested that notable changes in renal and liver laboratory safety data were reflective of changes in serum ferritin levels over the course of the study, particularly in the 10 and 15 mg/kg/day cohorts. Several patients with low baseline serum ferritin levels, or those who experienced rapid reductions in serum ferritin, had notable increases in ALT or serum creatinine levels that required dose modifications and/or interruptions; there was no clear relationship between the occurrence of these laboratory abnormalities and time on study. A risk of AEs, including renal disorder following over-rapid chelation, has also been identified in transfusion-dependent patients and particular attention is advised for monitoring of serum creatinine levels in patients who are receiving high doses of deferasirox with low rates of transfusion.^20^,^21^ These data suggest that appropriate target serum ferritin levels should be predefined and rapid iron depletion is to be avoided in order to prevent the occurrence of AEs, particularly because many patients may be asymptomatic. This can be achieved by closely monitoring body iron levels and adjusting deferasirox doses accordingly. Serial measurement of serum ferritin levels is a convenient and inexpensive tool that has been widely used for monitoring body iron levels and the efficacy of chelation therapy in various transfusion-dependent anemias.^10^,^20^ Although no conclusive mechanistic explanation for the increases in serum creatinine observed in this study is available yet, it is likely related to modified renal hemodynamics as a result of deferasirox therapy.^22^,^23^ Although not an integral part of the protocol in the present study, serum creatinine levels in a limited number of patients who were monitored after treatment returned to normal. In addition, data from transfusion-dependent patient populations have shown that renal and hepatic events are generally nonprogressive and return to within normal limits following drug discontinuation.^20^,^24^,^25^

The incidence of AEs, particularly in the core study, was slightly higher than that observed in patients with transfusional iron overload,^20^ although the lower number of patients included in this phase 1/2 trial should be considered. Our data suggest this difference may be related to the lower levels of iron burden in patients with HH, as well as lower rates of iron accumulation in the absence of ongoing red blood cell transfusions. This is supported by the higher rate of serum creatinine and ALT level increases in patients with lower serum ferritin levels or those with rapid reductions in serum ferritin following chelation therapy. Furthermore, as the patients in this study were generally asymptomatic, reported AEs may be comparatively higher due to the lack of medical conditions at baseline in the majority of patients, as opposed to other patient populations studied with additional underlying medical problems. The incidence of AEs during therapy with deferasirox may have implications for patient compliance and ultimately, the acceptance of iron chelation therapy for the treatment of patients with HH. In addition, the ease, effectiveness, and relatively low cost of phlebotomy therapy make it likely that phlebotomy will remain the treatment of choice for the vast majority of patients with HH.

Although phlebotomy and iron chelation therapy are effective for reducing iron burden in patients with HH, natural history studies of untreated C282Y homozygotes have demonstrated that progressive iron accumulation does not always occur.^26^–^29^ As such, further consideration should also be given to the identification of suitable candidates for therapy^30^ by monitoring serum ferritin levels prior to and during any subsequent treatment.

Some of the limitations of this study were that many of the patients had mild iron overload as assessed by serum ferritin levels, and a number of patients were treated with chelation therapy after having previously received phlebotomy. This was a phase 1/2 dose finding and safety assessment study of deferasirox in patients with HH, and as such the study involved a small number of patients. Therefore, further studies in larger numbers of patients with HH and assessments of the effects of *de novo* iron chelation therapy would be of value.

In conclusion, the findings reported here on the use of deferasirox to reduce iron in patients with nontransfusional iron overload due to HH are encouraging. Based on the safety and efficacy results, a starting dose of 10 mg/kg/day appears to be the most appropriate in this population; close monitoring of renal and hepatic function is required, especially in patients with lower body-iron burdens. Larger studies are warranted to more fully define the appropriate role of deferasirox for the treatment of selected patients.

## Abbreviations

AE: adverse event
ALT: alanine aminotransferase
AST: aspartate aminotransferase
C282Y: cysteine-to-tyrosine mutation at position 282
HH: hereditary hemochromatosis
ULN: upper limit of normal
